# Challenging Cognitive Demands at Work, Related Working Conditions, and Employee Well-Being

**DOI:** 10.3390/ijerph15122911

**Published:** 2018-12-19

**Authors:** Sophie-Charlotte Meyer, Lena Hünefeld

**Affiliations:** German Federal Institute for Occupational Safety and Health, D-44149 Dortmund, Germany; Huenefeld.Lena@baua.bund.de

**Keywords:** cognitive demands, occupational health, employee well-being, working conditions

## Abstract

In times of digitalized workplaces the extent of challenging cognitive demands at work is rising and employees increasingly have to manage new and unlearned tasks. Yet, these work characteristics have received little attention on how they relate to the worker’s well-being. Thus, we analyze associations between cognitive work demands—also in interaction with other job characteristics—and indicators of employee well-being. The analyses are based on the BIBB/BAuA Employment Survey 2018, a cross-section that is representative for the German working population and covers approximately 20,000 employed individuals. Ordinary least squares (OLS) regressions suggest that cognitive demands are associated with a higher probability of feeling fatigued. In contrast, the results with respect to the employees’ self-rated health status and job satisfaction are ambiguous, depending on which cognitive demand is considered. Overall, the findings indicate that cognitive demands might be related to both resource and demand, depending on the individual resources of employees.

## 1. Introduction

The world of work is undergoing permanent changes, which imply new challenges for organizations and individuals [1]. In globalized markets, organizations need to be more flexible, less hierarchical, and continually reorganizing to maintain competitiveness and prosperity. Furthermore, changes in the world of work are enabled and accelerated by digitization processes [2,3,4]. On the one hand, new technologies—such as information and communication technologies—are introduced as a strategy for adapting to constant market pressure. On the other hand, new technologies are again the basis for fundamental reorganization within organizations [5]. This results in a cycle of changes and the developments occur at an increased speed [3,4]. Overall, the world of work becomes more flexible, more unpredictable, and changes at an accelerated pace.

All these phenomena involve altered job demands for employees and lead to changes in job quality. Besides an intensification of work effort [6,7,8], as well as planning and decision-making demands [9,10,11], increases in cognitive and learning demands at work are discussed as outcomes of change in the working world [2,12,13]. Generating new knowledge, as well as problem solving, has become an integral part of employees’ work tasks [14,15]. Furthermore, employees have to acquire new skills constantly in order to adapt to rapidly changing demands at work [12]. Therefore, maintaining one´s skills has become more difficult due to increasing skill variety in recent years [11] and employees are more frequently confronted with tasks they have not learned or they are not familiar with. Coping with the new requirements and tasks is becoming increasingly relevant, not only for everyday working life but also for personal development, regarding competencies and skills learned in order to maintain employability [16,17,18].

In the past decades, research on the quality of work has often focused on work intensification and autonomy [19], showing that an appropriate balance between work requirements and autonomy is particularly important for a good quality of work, and in turn for employee well-being [20,21,22,23,24,25,26,27]. However, empirical studies on learning and cognitive demands are scarce and so far little is known about how these demands relate to the well-being of employees. The existing studies predominately focus on learning demands and point to an ambivalent picture regarding the relationship between these demands and employee well-being [13,28]. Moreover, the studies are based on non-representative survey data with small sample sizes, rendering it difficult to generalize the findings for the entire working population. There is also no distinct definition between learning or cognitive demands and both terms are largely used interchangeably. While both refer to confrontation with new tasks at work and the requirement to acquire new knowledge, learning demands can be understood as a superordinate term that includes cognitive demands [29]. In general, learning demands can be defined as demands which “require employees to acquire knowledge and skills that are necessary to perform their jobs effectively” [13]. Cognitive demands involve confrontation with new tasks, unpredictable developments, and solving routine problems [28]. Using this definition, it remains unclear whether an individual learning process is achieved or not and which demands contribute to the cognitive development of the employees [30].

In this study, we add to the existing literature and empirically explore the relationship between cognitive demands and employee well-being. The analyses are based on the German BIBB/BAuA Employment Survey 2018, a large representative cross-section providing recent data on the work and health situation of the working population in Germany (approximately 20,000 respondents). In a first step, we explore the determinants of cognitive demands in order to identify groups frequently facing cognitive demands at work. To measure cognitive demands, we considere three different variables: facing new tasks, improving work, and doing unlearned things. Analyzing the status quo is crucial in order to identify groups of workers with an increased need for additional training or assistance to cope with new requirements. This is of particular relevance as cognitive demands will likely become more prominent in the future and will gradually affect the whole working population. In a second step, we analyze the relationship between cognitive demands—also in interaction with other work demands—and employee well-being. We consider indicator variables for fatigue, self-rated health, and job satisfaction to measure employee well-being.

## 2. Work–Stress Theories and the Role of Cognitive Demands

Researchers developed different theoretical models to describe the relationship between different working conditions and employee health (e.g., Job Demands Control Model (JDC) [25], Job Demands-Resources Model (JD-R) [31], or Action Regulation Theory (ART) [32]. While these models consider various working conditions, only a few explicitly include cognitive demands. In the context of our study, two models are of particular importance. Firstly, we rely on the integrated model of psychosocial work characteristics and the consequences of job strain introduced by Glaser et al. [28]. Based on various theories and models [33,34,35] on the impact of working conditions on attitudes and health, the authors [28] developed a model embedding learning demands, work-related resources, and job stressors in order to predict processes of learning, performance, and health impairment. An important assumption of this model—in line with Karasek [25]—is that not all working conditions should be defined as job demands, regardless of their impact on employee well-being. When cognitive demands are predisposed as working conditions that trigger effort-driven processes and are thus associated with physical and psychological costs, the potential positive effects of cognitive demands for skill acquisition and performance are neglected. The absence of cognitive demands at work could also be negatively related to employee well-being and motivation [28]. Therefore, the authors combine the assumptions of different work-stress theories, including the challenge–hindrance framework that distinguishes between challenge and hindrance demands [36]. Hindrance demands are supposed to reduce personal growth and promote strain and health impairments [36,37]. Challenge demands also trigger strain, but they are also supposed to have a motivating effect and enable employees to learn and to further develop their own personality. The classification of job demands as challenge or hindrance demands depends on the individual assessment of employees. Therefore, the cognitive appraisals (challenge and hindrance appraisal) of individuals are the important explanatory mechanisms behind the positive and negative effects of job demands on a workers well-being [13,38,39,40]. In line with this framework, Glaser et al. [28] distinguish between beneficial learning (e.g., task variety and cognitive demands), work-related resources (e.g., autonomy and social support), and stressors or adverse conditions (e.g., overload and conflicts). The proposed model predicts a positive effect of learning demands on personality development and a negative effect of stressors or adverse conditions on health. Empirical analyses exploring this model indicate that problem solving and learning requirements are crucial for creativity and motivation. Other studies confirm these results. For instance, Prem et al. [13] find a significant relationship between learning demands and personal development. Personal development was in turn positively associated with vitality in this study. Furthermore, Crawford et al. [41] show that the correlation between work demands and engagement strongly depends on the specific type of work demand. Demands that were appraised by workers as hindrance were negatively associated with engagement and demands that were appraised by workers as challenges were positively associated with engagement. However, the study of Glaser et al. [28] also showed that learning requirements may be detrimental to health if accompanied by work overload. The authors thus demonstrate—in line with other studies [30,42]—the importance of the interaction of cognitive demands with other working conditions for employee well-being. Based on the model of Glaser et al. [28], we assume that cognitive demands might be both positively and negatively related to the personal development of employees, and in turn also to their attitudes and health. Furthermore, we assume that autonomy and work intensification moderate the effect of cognitive demands on employee well-being. 

Secondly, person–environment fit theories (P–E fit) are crucial to explain why cognitive demands at work could have different consequences for different groups of employees. All P–E fit theories assume that the extent to which people fit their work environments has significant consequences (e.g., with respect to their satisfaction, performance, stress, productivity, or turnover). A better fit is associated with better outcomes [43,44,45]. Moreover, Kristof-Brown and Guay [46] showed that stress is a consequence of a poor person–environment fit. The fit of the individual and the environment is determined by the fulfilment of needs resulting in favorable attitudes, such as job satisfaction or organizational commitment [47,48]. In addition, P–E fit is a reciprocal and ongoing process whereby individuals shape their environments and environments shape individuals [49]. Work environments are associated with cognitive demands to varying degrees. Furthermore, individuals also differ in terms of their needs at work and require different conditions in order to achieve favorable attitudes. In line with the P–E fit theories, we suppose that cognitive demands at work do not equally meet the needs of different employment groups. Consequently, we expect that the probability of perceiving cognitive demands at work as stressful varies across different groups of employees (e.g., with respect to different socio-demographic groups, such as gender or educational level).

## 3. Data and Methods

The analyses are based on the BIBB/BAuA Employment Survey 2018, a representative cross-sectional survey covering approximately 20,000 employed individuals in Germany who work at least 10 h per week and are at least 15 years old [50] (see Table A1 for an overview). The BIBB/BAuA Employment Survey is representative for the German labor force, including employed as well as self-employed individuals, and covers various occupational groups. For the analyses, we exclude individuals above the age of 65, as working individuals above that age (age 65 ≈ statutory retirement age in Germany) are a selective and highly heterogeneous group (e.g., self-employed, as well as individuals depending on additional income to increase their pension). Further, the analyses are restricted to individuals with valid data for the included variables. The analysis sample amounts to 18,554 individuals, although the number of observations varies slightly according to the analyses performed. The sample consists of 54.5% men and 45.5% women, with the majority (51.2%) being aged between 35–54 years (see Table A2 in Appendix A). Before the BIBB/BAuA Employment Survey 2018 has been carried out, it has been inspected and received a positive vote of the ethics commission.

### 3.1. Variables

The BIBB/BAuA Employment Survey 2018 includes three different variables indicating cognitive demands: facing new tasks, improving work, and doing unlearned things. We consider all of the three items in order to cover different dimensions of cognitive demands. Although the wording of the questions on cognitive demands is not identical, the indicators are still comparable to those used in previous studies [13,28]. The respondents were asked how often they face these demands during their work and the response scale was frequently, sometimes, rarely, never (see Table 1). In order to keep the analyses simple and reduce complexity, we recode the variables into indicator variables. For new tasks and improving work, these dichotomous variables equal 1 if someone reports to experience a cognitive demand frequently and 0 otherwise. As comparatively few individuals report doing unlearned things frequently, we collapse sometimes and frequently into one category for this variable.

A special feature of the BIBB/BAuA Employment Survey is that individuals who report frequently facing a specific job demand are subsequently asked whether or not they perceive this job demand as stressful. This allows us to take the individual assessment whether the specific cognitive demand is perceived as a resource or rather as a demand into account. For the variable “improve work”, the question whether it is stressful or not was not asked, as this question was not defined as a stressor due to its rather positive meaning.

Employee well-being is operationalized by three different outcomes. Work-related fatigue (yes = 1, no = 0) is based on the question of whether the respondent has frequently suffered from emotional exhaustion in the last 12 months during work, on working days, or both. A comparable dichotomous measure of fatigue is collected within the European Working Conditions Survey (EWCS) and has been previously used to study work-related differences in well-being [51]. Moreover, respondents were asked about their general state of health, which was recoded into an indicator variable for self-rated good health (excellent/very good/good = 1, not so good/poor = 0). Self-rated health has been a widely used measure and it was found that it likely considers chronic and acute illnesses [52,53]. Finally, we consider overall job satisfaction (by asking the question: “And now, all things considered, how satisfied are you with your work on the whole?”), recoded into an indicator variable (very satisfied/satisfied = 1, less satisfied/not satisfied = 0), as a measure for overall well-being. Single-item measures of global job satisfaction have been found to be as reliable as multiple-item measures [54].

As additional working conditions, we consider a measure for work intensity (by asking the question: “How frequently does it happen during your work that you have to work under great time pressure or pressure to perform?“), as well as an indicator for the respondent’s autonomy at work (by asking the question: “How frequently does it occur that you can plan and arrange your own work yourself?“). These variables equal 1 if the individuals report frequently facing the working conditions, and 0 otherwise (sometimes/rarely/never).

As control variables, we include gender, three age groups (15–34, 35–54, 55–65), three dummies for schooling (low, intermediate, high), four dummies for the occupational status, as well as five sector dummies (see Table A2 in Appendix A).

### 3.2. Methods

For ease of interpretation, we performed Ordinary Least Squares (OLS) regression analyses with robust standard errors. We thus applied linear probability models, given that the cognitive demands, as well as the health outcome measures, were coded as indicator variables. Coefficients may thus be interpreted as differences in the probability of the outcome variable—in this case facing a specific cognitive demand or a certain health condition. Given that linear and logistic models often render very similar results, we preferred this model, as this interpretation is much more intuitive and fits the research questions studied somewhat better compared to the interpretation of other methods (e.g., odds ratios derived from logistic regressions) [55]. In order to adjust for the violation of homoscedasticity, heteroscedasticity-consistent standard errors were applied.

In a first step, we explored the determinants of cognitive demands by regressing the cognitive demands on the control variables to get an idea of the groups frequently facing cognitive demands at work. Second, we explored whether, and to what extent, cognitive demands are related to employee well-being. We additionally exploited the information on whether or not the specific cognitive demand is perceived as stressful by restricting the analyses to those reporting to be frequently facing new tasks or doing unlearned things. In doing so, we aimed to make an effort in testing the P–E fit (see Section 2) and assess whether the cognitive demands are related to health or whether it depends on the individual’s characteristics. Moreover, these analyses enabled us to estimate the relationship between cognitive demands and health at the intensive margin, and thus mitigate the potential bias resulting from selection into the extent of facing cognitive demands. Previous studies suggest that cognitive demands might be harmful if they co-occur with work overload [28]. Therefore, in the final analyses we included interaction terms between one specific cognitive demand (i.e., doing unlearned things) and two working conditions (work intensity and autonomy) in the regressions of well-being. As a result, we were able to assess whether or not facing high work intensity or autonomy moderates the relationship between this cognitive demand and employee well-being. We focused on this cognitive demand as our analyses revealed that it is strongly related to adverse health or well-being, and, therefore, can be interpreted as a stressor. Work intensity and job autonomy were chosen, as according to common work–stress theories, their importance for employee well-being is well explored and widely accepted. Both the measures for cognitive demands and working conditions were operationalized as indicator variables, and thus the interaction terms between these two variables can be interpreted as follows: a positive interaction suggests that the working condition strengthens the association between the specific cognitive demand and employee well-being, while a negative interaction term mitigates the association.

## 4. Results

### 4.1. Distribution of Cognitive Demands

Table 1 summarizes the variables for cognitive demands studied in this paper. A vast majority reported to facing new tasks sometimes (39.9%) or even frequently (40.2%). Similarly, about three quarters of the respondents stated they were either sometimes or frequently required to improve procedures or try out something new. Doing unlearned things during work was less common; only 8.2% reported that their job frequently required doing things they had not learned or were not able to perform. With respect to facing new tasks at work, this applied to 18.3%. Among those doing unlearned things frequently, 42.16% perceived it as stressful.

### 4.2. Heterogeneity in Cognitive Demands Across Groups

As a first step, we explored the determinants of workers facing the three different types of cognitive demands at work by regressing the cognitive demands on socio-demographic characteristics (Table 2, Columns 1–3). Table 2 shows the results and each column presents the estimates of a separate regression model taking the different measures of cognitive demands as dependent variables. In general, women faced cognitive demands at work significantly less often as compared to male employees. For instance, the probability of facing new tasks at work is 7 percentage points lower for women. With respect to age, we found that middle-aged and older workers were significantly less likely to perform any of the cognitive tasks explored as compared to younger workers. High-educated individuals faced cognitive demands more often than low-educated, whereas the estimate for intermediate-educated individuals turns out to be significant for facing new tasks only. For new tasks, the educational differences in cognitive demands were most pronounced; high-educated individuals had a 19 percentage point higher probability to face new tasks at work compared to low-educated individuals. Regarding occupational status, white-collar employees, civil servants, and self-employed individuals are significantly more likely to experience cognitive demands at work as compared to blue-collar workers. Moreover, the results indicate that cognitive demands are most common within the manufacture sector, while they are less common in the service sector.

Interestingly, the results were opposed (Table 2, Columns 4 and 5) when we focused on the subjective perception of cognitive demands with respect to the question on whether it is stressful or not (given the respondent faced the specific cognitive demand frequently).; the probability of perceiving new tasks or doing unlearned things at work as stressful was significantly higher for women and older people as well as low-educated individuals.

### 4.3. Cognitive Demands and Well-Being

In the next step, we assessed whether cognitive demands were related to indicators of well-being (Table 3, Columns 1–3). Again, each column reports the estimates of separate regressions taking the different indicators of well-being as dependent variables. Overall the estimates revealed that cognitive demands played an important role for employee well-being—independent from their socio-demographic characteristics. With respect to fatigue, it turned out that all cognitive demands considered were associated with a higher probability of feeling fatigued. For instance, doing unlearned things sometimes or frequently was associated with a 10.6 percentage point higher probability of suffering from fatigue during work or on working days. Regarding overall health, the results varied across the different cognitive demands. While the probability of reporting to be in good health was not significantly related to facing new tasks at work, it was positively correlated with improving work. In contrast, doing unlearned things was associated with a reduced probability of reporting good health. Regarding job satisfaction, it turned out that on average, individuals facing new tasks and improving work frequently were more likely to be satisfied with their job. On the contrary, doing unlearned things was often associated with lower job satisfaction. The results indicate that cognitive demands might be related to both resource and demand.

The relationship between cognitive demands and well-being might be (partly) driven by whether or not individuals perceive the respective cognitive demand as stressful. For that reason, we now focus on individuals facing cognitive demands frequently and compare those perceiving it as stressful to those who do not regard it as a stressor (Table 3, Columns 4–6). As expected, the estimates became much larger. Perceiving it stressful to face new tasks or to do unlearned things at work was significantly associated with adverse health, e.g., with a higher probability of feeling fatigued but also with a lower probability of being satisfied with the job.

### 4.4. Cognitive Demands, Interactions with Other Working Conditions, and Well-Being

Table 4 reports the results of the interaction models with each column presenting the results of a separate regression. The models presented in Table 4 differ from the model estimated in Table 3, as the other cognitive demands (new tasks and improve work) are not included. For this reason, we also report the results for the relationship between unlearned things and the indicators for well-being where work intensity and autonomy have not been included (Table 4, Column 1, 3, 5). However, the estimates are quantitatively and qualitatively comparable to those presented in Table 4.

The interaction terms (Table 4, Column 2, 4, 6) revealed that work autonomy likely buffers the adverse relationship between cognitive demands (i.e., doing unlearned things) and employee well-being to some extent. While doing unlearned things was related to a 12.3 percentage point higher probability of feeling fatigued for those employees experiencing little autonomy at work, the association was reduced by 4.4 percentage points for those individuals reporting a high level of job autonomy. The same was also true with respect to overall health and job satisfaction. For instance, while doing unlearned things was related to a 5.8 percentage point lower probability of being in good health for individuals with a low level of job autonomy, the negative association was about half as strong for employees with a high level of autonomy. Regarding work intensity, the interactions turned out to be insignificant and quantitatively negligible.

## 5. Discussion and Conclusions

Cognitive Demands are an integral part of work environments nowadays. However, these demands have received little attention on how they relate to employee well-being. Based on the integrated model of psychosocial work characteristics and consequences of strain [28], we assumed that cognitive demands might be both positively and negatively related to the employees’ attitudes and health. Furthermore, based on P–E fit theories, we expected that the perception of whether cognitive demands are stressful or not would largely depend on socio-demographic and work-related characteristics. 

The theoretical assumptions were largely supported and the main result of our study is that cognitive demands play an important role in the workers’ well-being. Our analyses suggest that all cognitive demands considered are associated with a higher probability of feeling fatigued. However, with respect to self-rated overall health status and job satisfaction, the results are ambiguous, depending on the specific cognitive demand considered. On the one hand, improving work is positively related to good health and job satisfaction, while doing unlearned things is negatively associated with these outcomes. Therefore, the results indicate that cognitive demands might be related to both resource and demand—depending on the specific type of cognitive demand. These findings emphasize the immanent assumption of Glaser et al.’s [28] model that a fine-grained distinction of job demands is needed to analyze the associations between working conditions and the employees’ attitudes and health. Furthermore, the results strengthen the theoretical challenge–hindrance framework. Cognitive demands trigger strain, but they can also have a satisfying effect. That is because cognitive demands often involve task variation or learning, which likely improves the employees’ personal development and might thus be health-enhancing in the long run [19]. However, how the cognitive demands are designed seems to be crucial, and whether or not these demands co-occur with other job demands and if employees assess cognitive demands as stressful. While facing new tasks and improving work are to some extent positively related to well-being, doing unlearned things is consistently negatively related to employee well-being. In addition, perception of stress in relation to facing new tasks or to doing unlearned things at work is significantly associated with a higher probability of feeling fatigued, but also with a lower probability of being satisfied with the job. This result strengthens the importance of the challenge and hindrance appraisal as an explanatory mechanism for the relationship between cognitive demands and well-being. The challenge appraisal thus reflects the perception of situations enabling personal development. In contrast, the hindrance appraisal is related to individual frustration due to the prevention of the fulfilment of self-relevant goals [13,38,40]. The interaction analyses further reveal that autonomy might mitigate the negative association between doing unlearned things and well-being to some extent. In line with previous studies and theories, this finding further emphasizes the role of autonomy as an important resource to buffer stressors at work [34,56]. Overall, our findings support the idea that specific working conditions might be related to both demand and resource and that more research based on integrated models of different working conditions, including cognitive demands, are needed.

Moreover, our analyses on the determinants of cognitive demands reveal that different groups of employees face cognitive demands at work to varying degrees. A vast majority reported facing new tasks at work, while doing unlearned things during work was less common. This might partly be attributable to the relatively negative wording of this question (see Table 1). Moreover, the three variables are also different from a theoretical perspective; performing new tasks and improving procedures at work also refer to task variation, which might be interpreted as a resource, not only as a stressor. The analyses also indicate that the extent to which individuals perceive cognitive demands as stressful varies across different groups of employees. High-educated employees most frequently report facing cognitive demands as compared to low-educated employees. As expected, this suggests that knowledge-intensive occupations in particular are exposed to cognitive demands. In contrast, the probability of perceiving cognitive demands at work as stressful is significantly higher for low-educated individuals. This is in accordance with the assumptions derived from the P–E fit theories. Cognitive demands are an integral part of the work of high-educated employees and thus probably also a significant part of the satisfaction of needs. It can also be assumed that highly educated individuals are more likely to actively ask for new tasks to reach job satisfaction of needs. In addition, high-educated employees often dispose of more resources at work, such as a higher level of autonomy, as compared to low-educated employees [57]. Our findings emphasize that the match of individual needs and requirements in the workplace is crucial. Future research should focus on this in more depth in order to investigate the impact of different cognitive demands with regard to content and varying degrees of difficulty on the attitudes and health of different employment groups.

### 5.1. Limitations

Although this study is the first examining the relationship between cognitive demands and employee well-being based on a large data set representative for the German working population, there are some limitations that have to be acknowledged. First, the interpretation is limited due to the cross-sectional nature of the study. Thus, the analyses allow alternative explanations as we are not able to account for reverse causality or unobserved heterogeneity. Consequently, future research should elaborate on this, for example by replicating the analyses within a longitudinal study design. Second, all measures were based on self-reports from participants, raising the risk of overestimated results due to common method biases [58]. However, various authors point out that subjective views are certainly an important indicator of objective health-related outcomes [59]. Self-reports may not be too problematic when investigating interaction effects: Common method effects are likely attenuating rather than strengthening interactions [60]. Third, we used single items to measure cognitive demands. Although single item measures are found to be valid [55,61], studies and theories presented at the beginning of the paper suggest that cognitive demands might be a multi-dimensional concept on which future research should focus on. Finally, our analyses are based on the whole working population rendering knowledge on the relationship between cognitive demands and employee well-being in a general sense, which can be interpreted as a first step in discovering this issue. In order to better understand this relationship, future research should elaborate on the heterogeneity across groups, for example by performing subgroup analyses with respect to gender, age groups, and educational level, but also occupations. This is crucial in order to derive concrete recommendations for action.

### 5.2. Practical Implications

This study adds to the limited research on the relationship between cognitive demands and employee well-being. Our results indicate that cognitive demands are both stressors and resources. Considering the rise of new (communication) technologies [62], cognitive demands at work seem to be an important but widely neglected topic in modern societies. On the one hand, the results underline the beneficial effects of cognitive demands at work. Cognitive demands should be included in work tasks, giving employees the opportunity to improve their personal development. However, the cognitive demands should not over-strain employees. Organizations have the responsibility to design workplaces according to the needs of their employees. To ensure that the employer is informed about the cognitive demands of their employees, cognitive demands should also be included in the risk assessment and be a part of employee appraisals. Furthermore, organizations could create competence teams in which employees could exchange information on new challenges and learn from each other. Finally, organizations should offer additional training in order to support employees in developing individual coping strategies by considering the needs of different groups of employees.

## Figures and Tables

**Table 1:** Distribution of cognitive demands.

	*How Frequently Does It Happen during Your Work …*	Frequently	Sometimes	Rarely	Never	N	Stressful? (If Frequently)
New tasks	*… that you are faced with new tasks that you have to try to understand and become familiar with?*	40.2	39.9	15.0	5.0	19,509	18.3
Improve work	*… that you improve previous procedures or try out something new?*	29.2	44.8	17.6	8.4	19,485	n.a.
Unlearned things	*… that you are required to do things that you have not learned or do not have a mastery of?*	8.2	28.6	29.2	34.0	19,489	42.16

Source: Own calculations based on the BIBB/BAuA Employment Survey 2018, weighted results.

**Table 2:** Determinants of Cognitive Demands (OLS Regression).

				Demand is Stressful ^A^	
	New Tasks	Improve Work	Unlearned Things	New Tasks	Unlearned Things
**Gender**					
Men	Reference	Reference	Reference	Reference	Reference
Women	−0.0761 ***	−0.0427 ***	−0.0248 ***	0.0442 ***	0.0966 ***
	(0.0075)	(0.0071)	(0.0075)	(0.0090)	(0.0267)
**Age**					
15–34	Reference	Reference	Reference	Reference	Reference
35–54	−0.0225 *	−0.0207 *	−0.0276 **	0.0340 **	−0.023
	(0.0100)	(0.0096)	(0.0100)	(0.0104)	(0.0314)
55–65	−0.0521 ***	−0.0634 ***	−0.0684 ***	0.0988 ***	0.0901 *
	(0.0110)	(0.0104)	(0.0110)	(0.0127)	(0.0371)
**Schooling**					
Low	Reference	Reference	Reference	Reference	Reference
Intermediate	0.0565 ***	0.0153	0.0221	−0.0282	−0.0214
	(0.0114)	(0.0102)	(0.0115)	(0.0181)	(0.0427)
High	0.1924 ***	0.1154 ***	0.0714 ***	−0.0713 ***	−0.0916 *
	(0.0115)	(0.0104)	(0.0116)	(0.0176)	(0.0421)
**Occupational status**					
Blue-collar	Reference	Reference	Reference	Reference	Reference
White-collar	0.1193 ***	0.0985 ***	0.0551 ***	−0.0334	−0.047
	(0.0117)	(0.0102)	(0.0119)	(0.0186)	(0.0437)
Civil sevant	0.1847 ***	0.1498 ***	0.1793 ***	0.0385	0.0376
	(0.0181)	(0.0166)	(0.0181)	(0.0254)	(0.0610)
Self-employed	0.2003 ***	0.2207 ***	0.0273	−0.0706 **	−0.1430 *
	(0.0178)	(0.0167)	(0.0176)	(0.0222)	(0.0620)
**Sector**					
Public sector	Reference	Reference	Reference	Reference	Reference
Manufacture	0.0690 ***	0.0622 ***	0.0374 **	−0.0663 ***	−0.1013 *
	(0.0115)	(0.0110)	(0.0116)	(0.0135)	(0.0412)
Craft	−0.0033	−0.0444 ***	0.0336 *	−0.0964 ***	−0.1455 **
	(0.0151)	(0.0134)	(0.0151)	(0.0185)	(0.0526)
Service	−0.0332 ***	−0.0329 ***	−0.0180	−0.0710 ***	−0.0783 *
	(0.0099)	(0.0091)	(0.0098)	(0.0126)	(0.0375)
Other	−0.0352 **	−0.0085	0.0103	−0.0335	−0.0827
	(0.0135)	(0.0126)	(0.0135)	(0.0175)	(0.0478)
**Constant**	0.2719 ***	0.1964 ***	0.3209 ***	0.2486 ***	0.5362 ***
	(0.0173)	(0.0159)	(0.0175)	(0.0254)	(0.0627)
Adj. R^2^	0.0538	0.0388	0.0170	0.0345	0.0460
N	18,554	18,532	18,535	8235	1561

^A^ Corresponds to the subgroup of individuals who reported frequently facing the specific cognitive demand; Note: * *p* < 0.05, ** *p* < 0.01, *** *p* < 0.001; Robust standard errors in parentheses. Source: Own calculations based on the BIBB/BAuA Employment Survey 2018, unweighted results.

**Table 3:** Cognitive Demands and Well-Being (OLS Regression).

				Demand is Stressful ^A^		
	Fatigue	Overall Health	Satisfaction	Fatigue	Overall Health	Satisfaction
**Cognitive demands**						
New tasks	0.0289 ***	−0.0038	0.0138 **	0.1665 ***	−0.0867 **	−0.0826 **
	(0.0073)	(0.0056)	(0.0046)	(0.0383)	(0.0334)	(0.0299)
Improve work	0.0184 *	0.0147 **	0.0252 ***			
	(0.0077)	(0.0056)	(0.0045)			
Unlearned things	0.1064 ***	−0.0454 ***	−0.0510 ***	0.2227 ***	−0.1065 ***	−0.1312 ***
	(0.0071)	(0.0055)	(0.0046)	(0.0356)	(0.0287)	(0.0255)
Adj. R^2^	0.0339	0.0396	0.0149	0.1256	0.1282	0.0591
N	18,453	18,462	18,475	1146	1151	1151

^A^ Corresponds to the subgroup of individuals who reported frequently facing the specific cognitive demand; Note: * *p* < 0.05, ** *p* < 0.01, *** *p* < 0.001; Robust standard errors in parentheses; control variables included gender as well as dummies for age group, schooling, occupational status, and sector (see Table 2). Source: Own calculations based on the BIBB/BAuA Employment Survey 2018, unweighted results.

**Table 4:** Cognitive Demands, Interactions with Work Intensity and Autonomy, and Well-Being (OLS Regression).

	Fatigue	Fatigue	Overall Health	Overall Health	Satisfaction	Satisfaction
**Cognitive demands**						
Unlearned things	0.1148 ***	0.1234 ***	−0.0449 ***	−0.0578 ***	−0.0450 ***	−0.0649 ***
	(0.0069)	(0.0153)	(0.0053)	(0.0128)	(0.0045)	(0.0114)
**Other working conditions**						
Work intensity		0.1561 ***		−0.0713 ***		−0.0557 ***
		(0.0081)		(0.0064)		(0.0050)
Autonomy		−0.0282 **		0.0414 ***		0.0682 ***
		(0.0091)		(0.0077)		(0.0064)
**Interactions**						
Unlearned things x work intensity		0.0077		−0.0049		0.0031
		(0.0138)		(0.0106)		(0.0088)
Unlearned things x autonomy		−0.0437 **		0.0314 *		0.0310 **
		(0.0158)		(0.0133)		(0.0119)
Adj. R^2^	0.0324	0.0654	0.0397	0.0550	0.0123	0.0380
N	18,491	17,839	18,499	17,845	18,513	17,861

Note: * *p* < 0.05, ** *p* < 0.01, *** *p* < 0.001; Robust standard errors in parentheses; control variables included gender as well as dummies for age group, schooling, occupational status, and sector (see Table 2). Source: Own calculations based on the BIBB/BAuA Employment Survey 2018, unweighted results.

## Appendix A

**Table A1:** Overview of BIBB/BAuA Employment Survey.

	BIBB/BAuA Employment Survey
**Data owner**	German Federal Institute for Vocational Education and Training (BIBB) and the German Federal Institute for Occupational Safety and Health (BAuA)
**Survey**	Repeated cross-section, conducted every six years (comparable since 2006); Latest survey carried out in 2018
**Interview**	Telephone interviews (CATI) since 2006
**Sample**	Approximately 20.000 employees
**Target population**	Representative for the German working population; Includes individuals belonging to the labor force (having a paid work), aged 15 and over, with a regular work time of at least 10 h per week. Apprentices are excluded.
**Purpose of Data Collection**	Research according to the research programs of BIBB and BAuA; Aim to provide differentiated and representative information regarding the working population and jobs in Germany for quantitative employment and qualification research as well as for occupational health and safety reporting.
**Subjects**	Among others: Working conditions, work load, work-related health, main fields of responsibility, level of requirements, knowledge requirements, work requirements, need for advanced training, school education, vocational and advanced training, professional career, employment that is adequate to the vocational training, career changes, applicability of professional qualifications
**Further information**	https://www.bibb.de/en/12138.php

**Table A2:** Sample Statistics.

Variables	%
**Cognitive Demands**	
New tasks (frequently)	40.2
Improve work (frequently)	29.2
Unlearned things (frequently/sometimes)	36.8
**Gender**	
Men	54.5
Women	45.5
**Age**	
15–34	27.2
35–54	51.2
55–65	21.7
**Schooling**	
Low	22.4
Intermediate	38.1
High	39.6
**Occupational status**	
Blue-collar	18.7
White-collar	68.4
Civil sevant	5.4
Self-employed	7.5
**Sector**	
Public sector	25.0
Manufacture	20.6
Craft	11.7
Service	32.3
Other	10.4
**Work intensity** (frequently)	
Time pressure/pressure to perform	48.2
**Autonomy** (frequently)	
Arranging own work	65.4

Source: Own calculations based on the BIBB/BAuA employment survey 2018, weighted results.
